# Association of demographics, cardiovascular indicators and disability characteristics with 7-year coronary heart disease incident in persons with disabilities

**DOI:** 10.1186/s12889-023-16297-0

**Published:** 2023-07-17

**Authors:** Husheng Li, Yiyan Wang, Yan Xue, Yao Li, Hengjing Wu, Jing Wu

**Affiliations:** 1 School of Nursing, Shanghai University of Traditional Chinese Medicine, No. 1200, Cailun Road, Pudong New District, Shanghai, 201203 China; 2 Clinical Center for Intelligent Rehabilitation Research, Shanghai YangZhi Rehabilitation Hospital (Shanghai Sunshine Rehabilitation Center), School of Medicine, Tongji University, No. 2209, Guangxing Road, Songjiang District, Shanghai, 201619 China

**Keywords:** People with disability, Coronary heart disease, Epidemiology, Risk predictors, Cohort study

## Abstract

**Objective:**

Previous studies had demonstrated that disability increases mortality in patients with coronary heart disease (CHD). However, for people who had been disabled but do not have baseline cardiovascular disease, there is still limited data on how they might develop CHD. This study aimed to investigate the incidence and predictors of CHD in people with disabilities.

**Methods:**

We conducted a 7-year retrospective study utilizing data from the Shanghai Comprehensive Information Platform for Persons with Disabilities Rehabilitation. Subjects aged over 18 years with at least four annual complete electronic health records were included. The primary outcome was CHD, defined as ischemic heart disease or myocardial infarction. Kaplan–Meier analysis and log-rank tests were used to compare cumulative CHD for sub-populations, stratified by age, gender, and the classification of disabilities. Cox regression was used to identify the potentially important factors.

**Results:**

Out of 6419 persons with disabilities, 688 CHD cases (mean age 52.95 ± 7.17 years, male 52.2%) were identified, with a cumulative incidence of 10.72% and an incidence density of 15.15/1000 person-years. The incidence density of CHD is higher in the male gender, people over 45 years, and those with physical disabilities. Male (*HR* = 1.294, 95% *CI*, 1.111–1.506), hypertension (*HR* = 1.683, 95% *CI*, 1.405–2.009), diabetes mellitus (*HR* = 1.488, 95% *CI*, 1.140–1.934), total cholesterol (*HR* = 1.110, 95% *CI*, 1.023–1.204), and physical disabilities (*HR* = 1.122, 95% *CI*, 1.019–1.414) were independently associated with CHD.

**Conclusion:**

The findings indicate that the incidence of CHD differs across disability categories rather than the severity of disability. People with physical disabilities had significantly higher risks for the development of CHD. The underlying physiological and pathological factors need to be further studied.

## WHAT IS ALREADY KNOWN?

- CHD is one of the important causes of poor health condition, disability, and death. Disability caused by CHD is likely to be more lethal than disability resulting from other causes
- The associations between disease and disability are bidirectional. However, most of the existing studies provided information on the risks and prognostics of CHD, that treated disability as one of the outcomes.

## WHAT ARE THE NEW FINDINGS?

- In this 7-years cohort, we provide epidemiologic data of CHD secondary to disability status, including its incidence rate (15.15/1000 person-years) and risk factors. All subjects included in this study had been disabled and with no baseline CHD history, which was different from the study population in previous studies.
- The incidence rate of CHD differs across disability categories, rather than the severity of disability. Physical disabilities increase the risks of CHD, compared with other disability types. Cardio-metabolic risk factors remain a strong risk predictor of CHD among disabled population.

## WHAT DO THE NEW FINDINGS IMPLY?

- Our findings suggest that physical disability is associated with a higher risk of CHD than other types of disability, which is independent of traditional cardiovascular risk factors, such as age, male gender, and history hypertension. More detailed health monitoring and assessment strategies for the prevention of cardiovascular disease are needed for people with physical disabilities.

## Introduction

Disability refers to a persistent condition of having a body that functions abnormally, limits activities, or restricts participation [1]. It is estimated that over one billion individuals worldwide live with some form of disability [2]. This group of people is especially prone to health problems due to deficiencies in healthcare services and unequal access to medical care [3, 4]. Their diverse and heterogeneous nature further compounds the problem, leading to unmet health needs [5, 6]. Research shows that individuals with serious chronic illnesses have higher rates of disability. However, there is limited understanding of the emergence of chronic diseases in individuals who are already living with a disability.

Coronary heart disease (CHD) is a leading cause of disability and death worldwide, and its incidence is expected to rise due to the aging population [7, 8]. Several CHD risk prediction models have been developed based on longitudinal data from the general population [9–12]. Studies have demonstrated that the relationship between CHD and disability is bidirectional [13]. CHD patients with disabilities face higher mortality risks, and disability caused by CHD tends to be more lethal than disability caused by other factors. Despite these findings, most existing studies only focus on the risks and prognostics of CHD, and treat disability as a secondary outcome.

According to the Chinese national standard for *Classification and Grading Criteria of Disability* (GB/T 26341–2010) [14], there are four main types of disability: physical, visual, hearing & speech, and mental & intellectual. Each of these can be further categorized based on the degree of severity. Available studies have shown that people with disabilities are more likely to experience social isolation [15], have less healthy lifestyles [16], face barriers to accessing preventive healthcare [17, 18], and have higher all-cause and cardiovascular disease mortality rates [19–21]. Despite these findings, there has been limited research focused on investigating CHD incidence and risks in this population.

Our previous research [22] has revealed that people with disabilities, particularly those with physical disabilities, have a higher prevalence of hypertension. Based on this, we hypothesize that there may be differences in the incidence of CHD among individuals with disabilities. This study aims to retrospectively examine the incidence and potential predictors of CHD in disabled individuals using large-scale sample data. The results of this study will increase awareness of CHD in people with disabilities and assist healthcare practitioners in identifying CHD risks during health screenings for this population.

## Methods

### Data collection and ethics statement

This was a retrospective observational cohort study. The study data were obtained from the Shanghai Disabled Persons’ Rehabilitation Comprehensive Information Platform (SDPRCIP), which was established by the Shanghai Disabled Persons’ Federation (SDPF). SDPF has been providing a free health examination service for local persons with disabilities every year. Since 2012, the service has been designated to be implemented by Shanghai YangZhi Rehabilitation Hospital (Shanghai Sunshine Rehabilitation Center), and the relevant health examination records were uploaded by the hospital to the SDPRCIP for health follow-up. Examinees who voluntarily participated in this service have informed to the possible use of relevant health data for scientific research.

### Study population

**Fig. 1:**
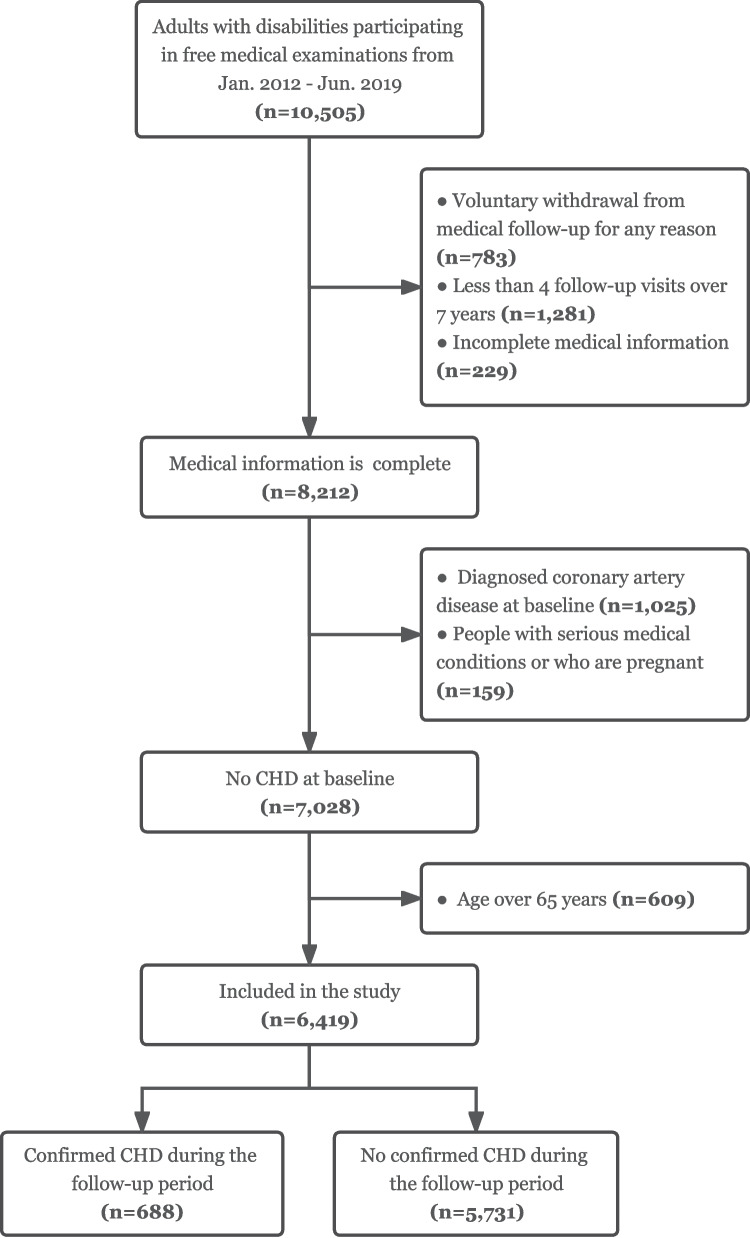
Consort flow diagram for participants included in the study. Note: CHD, coronary heart disease

People with disabilities who met the following criteria were included in this study: (i) age over 18 years old; (ii) attended at least 4 health examinations during the 7 follow-up years from 2012–2019 with complete electronic health records at each examination; (iii) attended the 2019 annual health examination. Those who did not fulfill the longitudinal follow-up or withdrawn for any reason, or those with one of the following conditions at baseline were excluded from the study: (i) confirmed CHD: the diagnosis was confirmed by a specialist physician with reference to WHO criteria; (ii) with other critical illnesses such as malignancy, organ failure, severe trauma; (iii) pregnant or lactating women, or those who are planning to become pregnant; (iv) elderly people aged 65 and above. Our final study sample consisted of 6419 persons with disabilities (Fig. 1).

### Assessment of new-onset CHD

The endpoint of this study was marked by the diagnosis of new-onset CHD during the follow-up period from January 2012 to June 2019. CHD is a heart condition caused by atherosclerosis leading to narrowing or blockage of the coronary arteries. This condition results in myocardial ischemia, hypoxia, or necrosis, so it is also referred to ischemic heart disease [23]. In the International Classification of Disease 10th revision code (ICD-10), the corresponding category codes are I20-I25 [24]. Therefore, in this study, the endpoint was defined as having a primary or any listed diagnosis of ICD-10 I20-I25 during the follow-up period.

### Study variables

In the collection of baseline characteristics, demographic variables include gender, age, occupation, educational background, etc.; medical history includes hypertension, dyslipidemia, diabetes, peripheral atherosclerosis, chronic kidney disease, etc.; disability history includes cause of disabilities, classification of disabilities, grade of disabilities, etc.; physical examination indicators include physical examination (height, weight, blood pressure, heart rate, eye examination, etc.), biochemical indicators (blood, urine indicators, etc.), electrocardiogram, imaging ultrasound, etc.

According to the Chinese national standard for *Classification and Grading Criteria of Disabilities* (GB/T 26341–2010) [14], the classification of disabilities included physical disabilities, visual disabilities, hearing & speech disabilities, or intellectual & mental disabilities. The grading criteria of disabilities was rated on a scale from level 1 to level 4, corresponding to very severe, severe, moderate, and mild disability, respectively.

### Statistical analysis

Descriptive analyses were compared between the CHD group and non-CHD group using the Pearson χ^2^ test for categorical variables, independent sample *t*-test for normally distributed data, and Mann–Whitney *U* test for non-parametric continuous data.

We used the Kaplan–Meier method to estimate the overall cumulative incidence of CHD from the baseline study visit onwards. The log-rank test was performed to compare a cumulative incidence curve for sub-populations, stratified by age, gender, and whether physical disabilities. Univariate and multivariate Cox proportional regression models were developed to calculate hazard ratios (HRs) and the corresponding 95% confidence intervals (CIs) attributable to potentially risk factors. Significant variables in baseline comparisons were entered into the multivariate model as covariates, including age, gender, body mass index (BMI), etc. The receiver operating characteristic (ROC) curves were also performed to further investigate the diagnostic capability of relevant independent risk factors in predicting the new-onset CHD in individuals with disabilities.

Applicable tests were two-sided, and *P*-value less than 0.05 was indicated statistically significant. All statistical tests were performed by using IBM SPSS Statistics version 26.0 (SPSS, Inc., Chicago, IL) and RStudio version 2022.02.3 Build 492 (http://www.rstudio.com) installed with survminer and survival packages. Data analysis was done in May 2022.

### Ethical considerations

The study protocol was reviewed and approved by the the Medical Ethics Committee of Shanghai Yangzhi Rehabilitation Hospital (Shanghai Sunshine Rehabilitation Center), School of Medicine, Tongji University (Approval No. YZ 2019–051). In view of the retrospective nature of the study, the Medical Ethics Committee of Shanghai Yangzhi Rehabilitation Hospital (Shanghai Sunshine Rehabilitation Center), School of Medicine, Tongji University waived the requirement for informed consent.

## Results

### Baseline characteristics of all participants with disabilities

Table 1 presents that a total of 6419 eligible individuals were included in the retrospective cohort. Age ranged from 18 to 64 years, with a mean age of 50.77 ± 9.88 years; 3046 (47.5%) were male and 3373 (52.5%) were female. Compared to those with non-CHD, patients with new-onset CHD were more likely to be older (mean age, 52.95 vs 50.51 years) and fatter (mean BMI, 24.33 vs 23.97), be male gender (52.2% vs 46.9%), had a higher prevalence of physical disabilities (61.2% vs 55.4%), hypertension (53.6% vs 34.9%), diabetes mellitus (19.2% vs 10.3%), and abnormal ECG (18.9% vs 10.9%). Grading of disabilities was not statistically significant in the comparison between groups. A number of metabolic biomarkers also differed significantly when compared between the two groups, including fasting blood glucose (FBG), total cholesterol (TC), total triglyceride (TG), serum urea (SU), and white blood count (WBC) (All *P* < 0.01).

**Table 1:** Baseline characteristics of all participants with disabilities prior to the CHD onset

Characteristics	Overall population *n* = 6419	CHD group *n* = 688	Non-CHD group *n* = 5731	*P*-value
Age, yrs	50.77 ± 9.88	52.95 ± 7.17	50.51 ± 10.13	< 0.001^*^
Male, *n* (%)	3046 (47.5)	359 (52.2)	2687 (46.9)	0.010^*^
Classification of disabilities, *n* (%)				< 0.001^*^
Physical disabilities	3594(56.0)	421 (61.2)	3173 (55.4)	
Visual disabilities	1346 (21.0)	160 (23.3)	1186 (20.7)	
Hearing & speech disabilities	370 (5.8)	30 (4.4)	340 (5.9)	
Intellectual & mental disabilities	1109 (17.3)	77 (11.2)	1032 (18.0)	
Whether physical disabilities, *n* (%)				0.004^*^
Physical disabilities	3594(56.0)	421 (61.2)	3173 (55.4)	
Other disabilities	2825 (44.0)	267 (38.8)	2558 (44.6)	
Grading of disabilities, *n* (%)				0.847
Very severe disability	502 (7.8)	50 (7.3)	452 (7.9)	
Severe disability	991 (15.4)	101 (14.7)	890 (15.5)	
Moderate disability	2224 (34.6)	240 (34.9)	1984 (34.6)	
Mild disability	2702 (42.2)	297 (43.1)	2405 (42.0)	
Comorbidities, *n* (%)				
Hypertension	2370 (36.9)	369 (53.6)	2001 (34.9)	< 0.001^*^
Diabetes mellitus	725 (11.3)	132 (19.2)	593 (10.3)	< 0.001^*^
Chronic kidney disease	901 (14.0)	107 (15.6)	794 (13.9)	0.223
Fatty liver disease	600 (9.3)	54 (7.8)	546 (9.5)	0.166
Abnormal ECG, *n* (%)	756 (11.8)	130 (18.9)	626 (10.9)	< 0.001^*^
Heart rate, beats/min	78.15 ± 10.23	78.53 ± 11.51	78.10 ± 10.07	0.351
BMI, kg/m^2^	24.01 ± 3.54	24.33 ± 3.52	23.97 ± 3.54	0.011^*^
Overweight, *n* (%)	3072(47.9)	349(50.7)	2723(47.5)	0.215
Blood pressure, mmHg				
Systolic	132.55 ± 20.23	135.3 ± 20.83	132.22 ± 20.13	< 0.001^*^
Diastolic	78.77 ± 12.51	80.38 ± 12.82	78.57 ± 12.46	< 0.001^*^
Metabolic biomarkers				
FBG, mmol/L	5.49 ± 1.44	5.84 ± 1.93	5.45 ± 1.36	< 0.001^*^
TC, mmol/L	4.78 ± 0.92	4.93 ± 0.96	4.76 ± 0.91	< 0.001^*^
TG, mmol/L	1.25 (0.89,1.84)	1.38 (1.01,1.99)	1.24 (0.88,1.83)	< 0.001^*^
Alb, g/L	43.58 ± 2.30	43.48 ± 2.27	43.59 ± 2.31	0.214
Glo, g/L	28.72 ± 3.72	28.75 ± 3.63	28.72 ± 3.74	0.819
UA, mol/L	313.80 (262.00, 373.83)	323.15 (266.20, 376.55)	312.80 (261.20, 373.70)	0.102
SCr, μmol/L	63.43 ± 22.93	63.13 ± 17.61	63.46 ± 23.49	0.717
SU, mmol/L	4.90 (4.20, 5.80)	5.00 (4.40, 5.90)	4.9 (4.20, 5.80)	0.001^*^
Hb, g/L	137.83 ± 16.15	136.73 ± 15.43	137.97 ± 16.24	0.059
RBC, 10^12^/L	4.71 ± 0.47	4.70 ± 0.48	4.72 ± 0.47	0.293
WBC, 10^9^/L	6.53 ± 1.67	6.73 ± 1.69	6.50 ± 1.67	0.001^*^
PLT, 10^9^/L	204.58 ± 55.82	208.39 ± 57.22	204.13 ± 55.64	0.058

Notes: Data are shown as means ± standard deviation, median (interquartile range), or numbers (percentages)

*ECG* Electrocardiogram, *BMI* Body mass index, *FBG* Fasting plasma glucose, *TC* Total cholesterol, *TG* Total triglyceride, *Alb* Albumin, *Glo* Globulin, *UA* Uric acid, *SCr* Serum creatinine, *SU* Serum urea, *Hb* Hemoglobin, *RBC* Red blood count, *WBC* White blood count, *PLT* Platelet count

^*^ = statistically significant

### Longitudinal association between baseline variables and incident CHD at follow-up

**Fig. 2:**
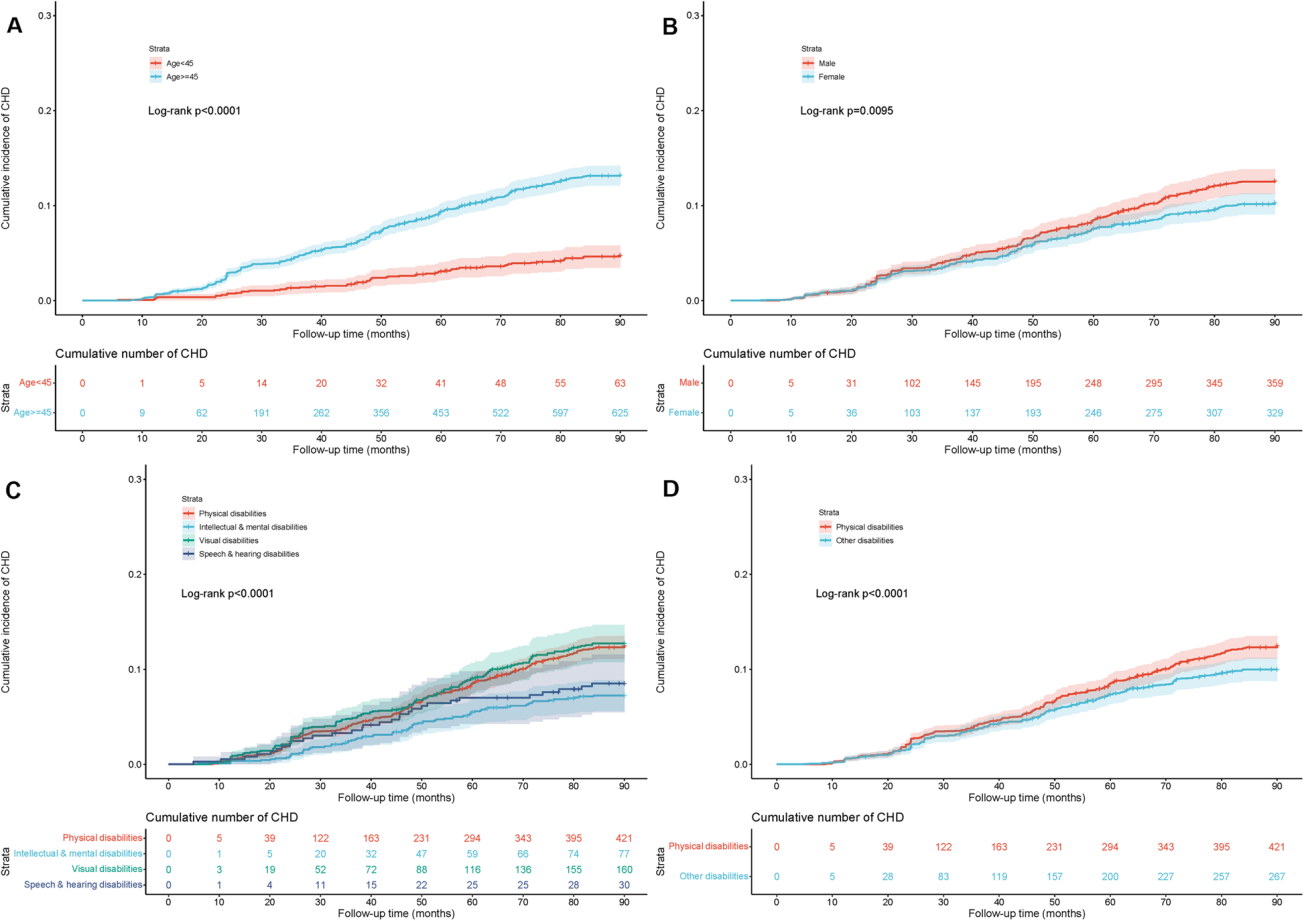
Kaplan–Meier curves for an overall cumulative incidence of CHD and number at risk, stratified by (**A**) age, **(B**) gender, (**C**) the classification of disabilities, and (**D**) whether physical disabilities. The shaded area indicates the range of 95% confidence intervals for the corresponding cumulative incidence curve. *P*-value indicates the significance level from the comparison of incidence curves using the Log-rank test

During a median follow-up period of 84.9 months, a total of 688 cases with incident CHD were identified, with a cumulative incidence of 10.72% and an incidence density of 15.15/1000 person-years. As displayed in Table 2, the incidence population consisted of 359 males with an incidence density of 16.73/1000 person-years and 329 females with an incidence density of 13.73/1000 person-years. Moreover, the incidence density of CHD is higher in people with physical disabilities (16.59 *vs*. 13.32 per 1000 person-years) and those over 45 years (17.60 *vs.* 6.35 per 1000 person-years). The Kaplan–Meier curves for an overall cumulative incidence of CHD and the number at risk are shown in Fig. 2, stratified by (A) age, (B) gender, (C) the classification of disabilities, and (D) whether physical disabilities. Analysis showed significant differences in the cumulative incidence curves for all subgroups (Log-rank, *P* ≤ 0.0095).

**Table 2:** Incidence of CHD in the disabled population according to baseline

Variables	Cases (*n*)	Incidence (%, 95% *CI*)	Incidence Density (Per 1000 Person-Years)	Log-rank	*P*-value
Overall	688	10.72 (9.96–11.38)	15.15		
Stratified by age				65.23	< 0.0001^*^
18 ~ < 45 yrs	63	4.64 (3.52–5.76)	6.35		
45 ~ < 65 yrs	625	12.35 (11.44–13.26)	17.60		
Stratified by gender				6.73	= 0.0095^*^
Male	359	11.79 (10.64–12.94)	16.73		
Female	329	9.75 (8.75–10.75)	13.73		
Stratified by classification of disabilities				23.90	< 0.0001^*^
Physical disabilities	421	11.71 (10.66–12.76)	16.59		
Visual disabilities	160	11.89 (10.16–13.62)	16.95		
Hearing & speech disabilities	30	8.11 (5.33–10.89)	11.41		
Intellectual & mental disabilities	77	6.94 (5.44–8.44)	9.65		
Stratified by whether physical disabilities				7.94	= 0.0048^*^
Physical disabilities	421	11.71 (10.66–12.76)	16.59		
Other disabilities	267	9.45 (8.37–10.53)	13.32		

*Note*: ^*^ = statistically significant

### Predictors of new-onset CHD in people with disabilities

**Fig. 3:**
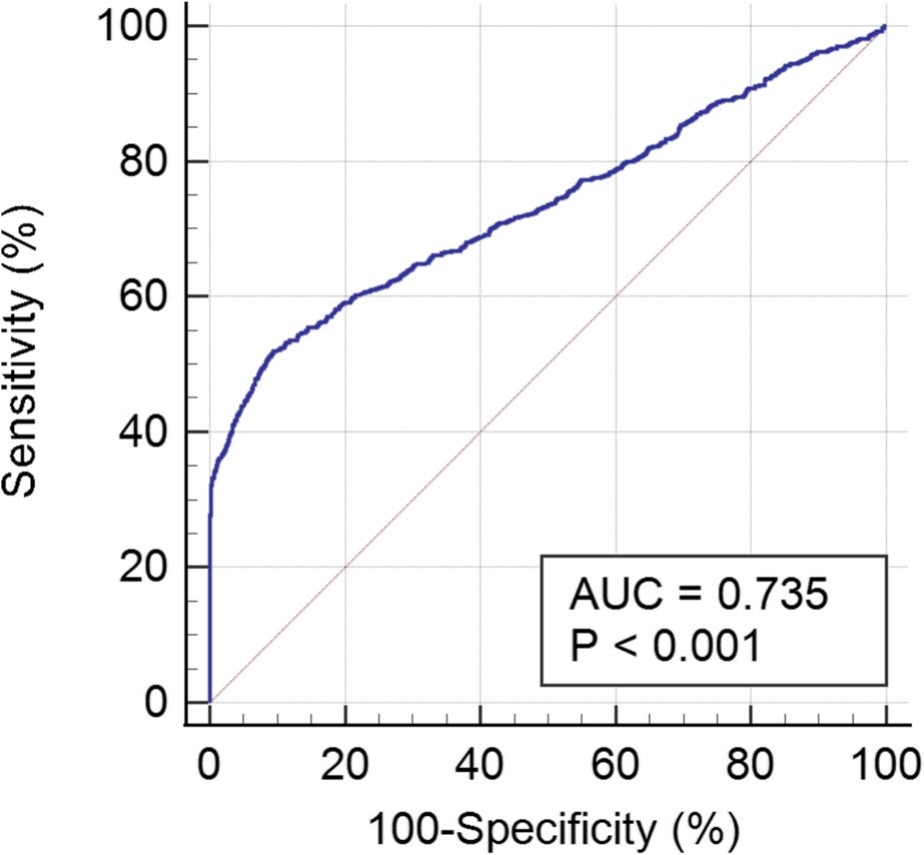
ROC curve of the independent risk factors in predicting the new-onset CHD in individuals with disabilities. The area under the curve (AUC) value ranges from 0.5 to 1.0, with near 1.0 indicating perfect predictive ability. The horizontal axis shows false positive rate, and vertical axis shows true positive rate

Cox proportional-hazards regression model (Table 3) was further conducted to examine the risk factors and interaction terms associated with new-onset CHD. The age-adjusted Cox analysis revealed that male gender (*P* = 0.005), BMI (*P* = 0.02), FBG (*P* < 0.001), TC (*P* < 0.001), hypertension (*P* < 0.001), diabetes mellitus (*P* < 0.001), and physical disabilities (*P* = 0.002) significantly associated with CHD risks. Furthermore, the multivariate Cox model demonstrated that independent predictive factors for CHD in the disabled population include male gender (*HR* = 1.294, 95% *CI*, 1.111–1.506), hypertension (*HR* = 1.683, 95% *CI*, 1.405–2.009), diabetes mellitus (*HR* = 1.488, 95% *CI*, 1.140–1.934), TC (*HR* = 1.110, 95% *CI*, 1.023–1.204), and being physical disabilities (*HR* = 1.122, 95% *CI*, 1.019–1.414). We also established the ROC curve using the independent risk factors from the multivariate Cox regression. It was shown that the risk score had better predictive power (AUC = 0.735) (Fig. 3).

**Table 3:** Cox regression model for the risk of CHD in the disabled population

Variables	Age-adjusted *HR* (95% *CI*)	*P*-value	Multivariable adjusted *HR* (95% *CI*)	*P*-value
Age	1.021 (1.011–1.030)	< 0.001^*^	1.011 (1.002–1.018)	0.017^*^
BMI	1.027 (1.006–1.049)	0.020^*^	0.995 (0.973–1.018)	0.659
Heart rate	1.004 (0.997–1.011)	0.285	1.000 (0.992–1.007)	0.865
FBG	1.124 (1.087–1.163)	< 0.001^*^	1.049 (0.998–1.104)	0.061
TC	1.191 (1.102–1.287)	< 0.001^*^	1.110 (1.023–1.204)	0.012^*^
Gender				
Female	Reference	–	Reference	–
Male	1.119 (1.020–1.316)	0.005^*^	1.294 (1.111–1.506)	0.001^*^
Whether physical disabilities				
Other disabilities	Reference	–	Reference	–
Physical disabilities	1.274 (1.168–1.649)	0.002^*^	1.122 (1.019–1.414)	0.009^*^
Hypertension				
No	Reference	–	Reference	–
Yes	2.064 (1.777–2.398)	< 0.001^*^	1.683 (1.405–2.009)	< 0.001^*^
Diabetes mellitus				
No	Reference	–	Reference	–
Yes	1.875 (1.434–2.188)	< 0.001^*^	1.488 (1.140–1.934)	< 0.001^*^

*Notes*: *HR* Hazard ratio, *CI*, Confidence interval, *BMI* Body mass index, *FBG* Fasting plasma glucose, *TC* Total cholesterol

^*^ = statistically significant

## Discussion

From what we know, there are limited substantial longitudinal population-based surveys to explore the association evidence between disability and CHD, particularly in Asian countries. The SDPRCIP is a government initiative focused on the Shanghai Disability Health Survey. The platform is dedicated to collecting authentic and reliable data, offering a rare chance to perform a retrospective cohort. In this study, we provide epidemiologic data of CHD secondary to disability status, including its incidence rate and risk factors. Notably, the incidence rate of CHD differs across disability categories, rather than the severity of disability. Compared with other disability types, subjects with physical disabilities may had significantly higher risk for the development of CHD.

The incidence rate of CHD in this study was 15.15/1000 person-years for people with disabilities, while it was 16.59/1000 person-years for those with physical disabilities. This result far exceeds the 5.57–6.37/1000 person-years from cohort studies in the general Chinese population [25, 26]. Consistent with the previous studies, our results demonstrated that people with disabilities have a higher risk of CHD than their healthy peers. Plichart et al. [20] revealed that subjects with moderate or severe disabilities have almost double the risk of CHD over 6 years, independent of major cardiovascular risk factors and other potential confounders. There are significant differences in the risk profiles stratified by disability types, subjects with visual disabilities and physical disabilities were more likely to incident CHD over time than those with hearing & speech disabilities, and intellectual & mental disabilities. However, the possible outcome bias caused by aging-induced degenerative visual disabilities and youth-led intellectual & mental disabilities should not be ignored.

By multivariable-adjusted Cox models, people with physical disabilities showed significantly higher incidence risk of CHD than other disability types. One of the possible explanations might be the mobility restrictions. It is established that physical inactivity, also known as sedentary lifestyle, is one of the major risk factors of CHD [27]. In addition to activity limitations and self-care deficits, people with physical disabilities are vulnerable to psychological stress and discrimination and benefit less from rehabilitation and health care services [3, 5, 15].

Existing evidence, while limited, supports that individuals with long term physical disability have been shown to be at higher risk of vascular comorbidities such as stroke compared to the general population [28, 29]. Growing experimental evidence suggested that endothelial cell dysfunction could be induced by the constantly exposure to low tensile and fluid shear stress [30–32]. Such hemodynamic conditions are more common in people with leg amputation. In addition to the psychological stress and patients’ deviant behaviors, we assumed that physical related hemodynamic abnormalities might be the underline risks of cardiovascular diseases.

Based on the results, BMI, FBG, TC, hypertension, and diabetes mellitus significantly increase the risk of CHD in people with disabilities. It is affirmable that important risk predictors for cardiovascular disease in people with disabilities are also accompanied by metabolic symptoms based on insulin resistance [33, 34]. It leads to CHD as a result of the interaction between abnormal lipid metabolism, hypertension, and hyperglycemia, which are mutually reinforcing. For people with disabilities, it is apparent that improving modifiable clinical risk factors (e.g., hypertension, diabetes, hyperlipidemia, etc.) can similarly reduce new-onset CHD.

### Strengths and limitations

The present study includes population-based data with representative sample size, thereby allowing for an accurate evaluation of the association between disability and CHD after a long period of follow-up, and the results are largely convincing. To our knowledge, this is the first time that the incidence density of CHD in China's disabled population has been documented.

However, several limitations should be considered. We recognize that individuals with severe disabilities may face mobility restrictions that prevent them from participating in health survey and other aspects of the study. As a result, the inclusion of these individuals may be constrained, leading to potential selection bias in the findings. Additionally, due to the limitations of the raw data, we were unable to assess important lifestyle factors such as drinking, smoking, and diet, as well as psychological perceptions among patients with disabilities. Moreover, although abdominal circumference and waist-to-hip ratio are known to be better predictors of long-term cardiovascular events than BMI, these measures were not included in the analysis. Therefore, further basic research and prospective cohort studies are needed to gain a better understanding of the underlying relationship between disability and cardiovascular disease.

## Conclusions

Our population-based, retrospective cohort study with a 7-year follow-up demonstrated that CHD is more prevalent in people with disabilities who are male and older than 45 years. Hypertension, diabetes mellitus, total cholesterol, and physical disabilities can be independent predictors of CHD in the disabled population. Specifically, the incidence of CHD is significantly higher in people with physical disabilities than in people with other classifications of disabilities. Consequently, there is a need for cardiovascular health screening among people with physical disabilities for early detection and intervention to reduce the occurrence and progression of an adverse cardiovascular event.

## Data Availability

The datasets generated in this study are not publicly accessible due to privacy and confidentiality reasons but are available from the corresponding author on reasonable request.

## Abbreviations

*Alb*: Albumin
*AUC*: The area under the curve
*BMI*: Body mass index
*CHD*: Coronary heart disease
*CIs*: Confidence intervals
*ECG*: Electrocardiogram
*FBG*: Fasting blood glucose
*Glo*: Globulin
*Hb*: Hemoglobin
*HRs*: Hazard ratios
*PLT*: Platelet count
*RBC*: Red blood count
*ROC*: The receiver operating characteristic
*SCr*: Serum creatinine
*SDPF*: Shanghai Disabled Persons’ Federation
*SDPRCIP*: Shanghai Disabled Persons’ Rehabilitation Comprehensive Information Platform
*SU*: Serum urea
*TC*: Total cholesterol
*TG*: Total triglyceride
*UA*: Uric acid
*WBC*: White blood count
